# Analysis of Electrophysiological Activation of the Uterus During Human Labor Contractions

**DOI:** 10.1001/jamanetworkopen.2022.14707

**Published:** 2022-06-02

**Authors:** Alison G. Cahill, Zichao Wen, Hui Wang, Peinan Zhao, Zhexian Sun, Alan L. Schwartz, Yong Wang

**Affiliations:** 1 Department of Women’s Health, Dell Medical School, University of Texas at Austin, Austin; 2 Department of Obstetrics and Gynecology, Washington University School of Medicine in St Louis, St Louis, Missouri; 3 Department of Pediatrics, Washington University School of Medicine in St Louis, St Louis, Missouri

## Abstract

This cohort study uses electromyometrial imaging to examine the underlying electrophysiological origins of human labor at the myometrium level.

## Introduction

During pregnancy, the human uterus expands up to 500-fold, remains quiescent for 9 months to support fetal development, produces forceful contractions at term to expel the fetus, and then returns to its prepregnancy state.^1^ We have a limited understanding of how the uterus accomplishes these feats. For example, we lack answers to basic questions about labor, such as where contractions initiate, how fast contractions propagate, which regions of the uterus are active during contractions, and how these measures change as labor progresses. The recently developed electromyometrial imaging (EMMI) technology^2^ allows for the quantitation of several new parameters (eg, uterine isochrone maps, 3-dimensional [3-D] uterine activation, and maximal activation ratio [MAR]). This study was performed to examine the underlying electrophysiological origins of human labor at the myometrium level.

## Methods

**Figure 1.**
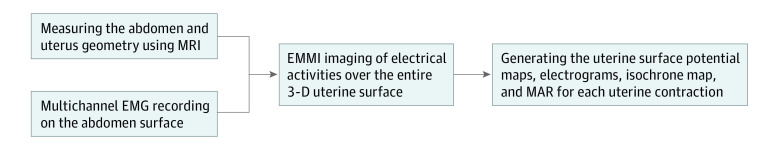
Pipeline of the Clinical Electromyometrial Imaging (EMMI) System Magnetic resonance imaging (MRI) is performed to generate the patient-specific abdomen and uterus geometry at 36 to 38 weeks of gestation. The patient wears up to 192 MRI-compatible markers during the MRI scan. Abdomen surface electromyograms (EMGs) are simultaneously recorded from up to 192 electrodes placed at the same positions as the MRI markers. EMMI software combines the abdomen-uterus geometry and abdomen surface EMGs to reconstruct the electrical activities over the entire 3-dimensional (3-D) uterine surface. For each uterine contraction, EMMI will generate uterine surface potential maps with high spatial-temporal resolution, uterine surface electrograms, the chronological sequence of electrical activations across the entire uterine surface (isochrone map), and the maximal activation ratio (MAR).

To explore term human labor with new EMMI-derived electrophysiological indices of uterine activity, we performed a prospective cohort study between August 9, 2017, and June 21, 2021, in an academic, tertiary care center in the Midwest US. This study was approved by the Washington University Institutional Review Board. A total of 18 women with uncomplicated singleton pregnancies were enrolled and signed the informed consent documents. Patients with exposure to medications known to affect uterine contractility, major fetal anomalies, prior spontaneous preterm birth, or contraindications for magnetic resonance imaging were ineligible. Participants at 36 to 38 weeks’ gestation underwent magnetic resonance imaging to obtain patient-specific uterine geometry. Once the patient was in active labor (≥5 cm dilated with regular contractions), up to 192 electrodes, placed on the patient’s abdomen and back, recorded body surface potentials for approximately 1 hour. EMMI data acquisition, data processing, and the resultant parameters are described (Figure 1). The primary EMMI outcome was MAR, the percentage of myometrium that is electrically activated during a uterine contraction. Secondary EMMI outcomes included the isochrone map, describing the detailed 3-D activation pattern. The 2-sided *F* test was performed to compare the variance of EMMI-derived electrophysiological indices. *P* < .05 was considered significant.

## Results

**Figure 2.**
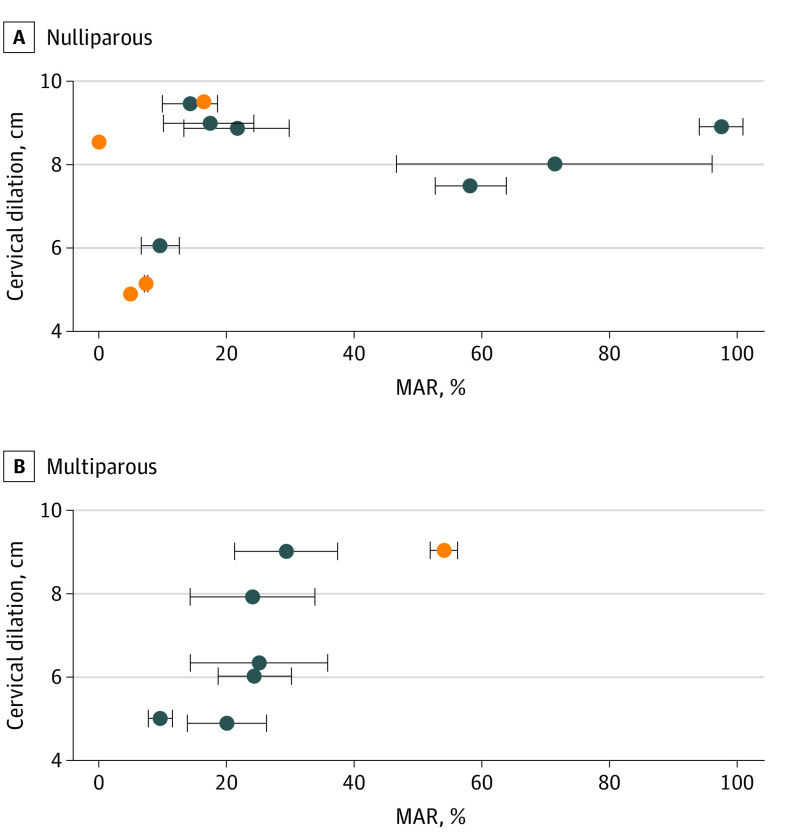
Primary Electromyometrial Imaging (EMMI) Outcome: Maximal Activation Ratio (MAR) A, MAR distribution in 11 nulliparous patients was displayed with respect to the cervix dilation measured at the end of EMMI. B, MAR distribution in 7 multiparous patients. The mean (SD) MAR values of all EMMI contractions from each patient are indicated by the circles and the error bars. The orange circles indicate the patients with less than 3 observed contractions during EMMI.

The study population included 11 nulliparous patients (mean [SD] age at delivery, 27.4 [5.5] years; mean [SD] body mass index at last prenatal visit, 30.1 [3.5] [calculated as weight in kilograms divided by height in meters squared]; mean [SD] birth weight, 3347 [196] g) and 7 multiparous patients (mean [SD] age at delivery, 24.9 [4.3] years; mean [SD] body mass index at last prenatal visit, 29.7 [3.8]; mean [SD] birth weight, 3414 [334] g). Among the 18 patients, the mean (SD) MAR in 11 nulliparous patients showed higher variance compared with the MAR in 7 multiparous patients (28.9% [31.9%] vs 26.6% [13.6%]) by the *F* test (ratio of variance, 5.5; 95% CI, 1.0-22.4; *P* = .04). (Figure 2).

## Discussion

In this study, we found that the correlation pattern of the EMMI-derived uterine activation index, MAR, and cervical dilation of the nulliparous group was more heterogeneous than that of the multiparous group. The most common methods of monitoring uterine contractions clinically during labor are tocodynamometry and intrauterine pressure catheters, neither of which provide spatial information, which can be observed by EMMI.^3,4^ The uterine magnetic activity has been measured with a superconducting quantum interference device from the anterior abdominal region.^5^ However, a superconducting quantum interference device requires installation in a magnetically shielded room, which significantly limits its wide application in clinical practice. In comparison, the uterine electrical activities can be reliably and cost-effectively measured using bioelectrodes in clinical environments, which enable EMMI to “see” and quantify the uterine contractions in 3-D noninvasively for each patient during labor.^6^ This electrophysiological difference underlying uterine contractions may indicate the existence of myometrial memory, leading to more rapid labor progression of multiparous patients than nulliparous patients. In the short term, EMMI may facilitate translational studies aimed at defining the mechanisms underlying normal human labor.

The major limitations of this study are the cost and accessibility of EMMI and the small patient cohort. We are developing a low-cost, portable, bedside EMMI system that does not rely on magnetic resonance imaging and can be applied to a larger patient cohort in clinical trials aimed at testing interventions to estimate when women experiencing contractions will deliver, how long labor will take, or which women will require intervention to prevent labor arrest, preterm birth, and postpartum hemorrhage.
